# Differential enrichment of key bacterial taxa in the rhizosphere of naturally growing and artificially restored *Kandelia obovata* forests

**DOI:** 10.1038/s41598-026-39157-4

**Published:** 2026-03-01

**Authors:** Shouji Gong, Riming Wang, Xiaokui Xie, Xiujian Li

**Affiliations:** 1 College of Food Engineering, Beibu Gulf University, Qinzhou, 535011 China; 2 Guangxi Key Laboratory of Marine Environmental Change and Disaster in Beibu Gulf, Beibu Gulf University, Qinzhou, 535011 China

**Keywords:** Bacterial community structure, Mangrove forest, *Kandelia obovata*, Artificial restoration, Ecology, Ecology, Ocean sciences

## Abstract

The structure of the soil bacterial community is crucial for maintaining ecosystem balance and facilitating material transformation in mangrove ecosystems. The large-scale destruction of mangroves directly impacts soil bacterial processes, potentially leading to ecosystem degradation. This study employed Illumina NovaSeq high-throughput sequencing to investigate the rhizosphere bacterial community of *Kandelia obovata* seedlings in both natural and artificially restored forests. Although alpha and beta diversity analyses revealed that the overall bacterial community structure was not significantly altered by artificial restoration, significant shifts in the abundance of specific bacterial genera were identified. A substantial proportion (86.1%-92.6%) of bacterial sequences remained unclassified at the genus level. Distinct dominant genera were observed across different groups: the well-grown artificial group (treat-k) was enriched with *Sulfurovum*, *Actibacter*, and *Desulfatiglans* (5.09%, 2.18%, and 1.82%, respectively), while the poorly-grown artificial group (treat-s) was characterized by *Ignavibacterium*, *Prolixibacter*, and *Woeseia* (2.10%, 1.21%, and 1.06%, respectively). The natural group was dominated by *Woeseia*, *Desulfatiglans*, and *Halioglobus* (1.56%, 1.53%, and 1.11%). Statistical analysis further confirmed that the abundance of several genera, including *Ignavibacterium*, *Prolixibacter*, and *Haliangium* differed significantly (*p* < 0.05) between the poorly-grown group (treat-s) and the better-grown groups (treat-k and natural). In conclusion, while artificial restoration did not restructure the rhizosphere bacterial community at a global level, it selectively shaped the microbial assemblage by enriching specific bacterial taxa, which might play a crucial role in determining the growth status of *Kandelia obovata* during restoration.

## Introduction

Mangrove forests, which thrive in tropical and subtropical intertidal zones, are essential coastal ecosystems that provide various services, including shoreline stabilization, carbon sequestration, and nursery habitat for marine life^1^. However, these ecosystems are currently facing unprecedented threats from human activities and climate change, resulting in widespread degradation and loss globally^2^. In response to these challenges, artificial restoration has emerged as a crucial strategy for rehabilitating damaged mangrove habitats.

The success of mangrove restoration is inextricably linked to the soil microbiome, particularly the rhizosphere bacterial communities. These microorganisms serve as the engine of nutrient cycling, facilitating critical processes in mangrove sediments, such as sulfur oxidation, sulfate reduction, and the decomposition of organic matter^3^. Furthermore, bacteria play a crucial role in mitigating pollutants, including hydrocarbons and microplastics, thus enhancing the resilience of mangrove ecosystems^4,5^. More importantly, specific functional groups, such as nitrogen-fixing and phosphorus-solubilizing bacteria, directly promote plant growth and health^6^. Consequently, the structure and function of the rhizosphere bacterial community are increasingly recognized as key indicators of mangrove ecosystem health and restoration efficacy.

While numerous studies have documented shifts in sediment microbial communities following mangrove restoration^7^; ^8^, a significant knowledge gap persists. Most research has concentrated on comparing restored sites with degraded states or on the temporal succession of communities. However, the comparative analysis of rhizosphere bacterial community structures in artificially restored mangroves versus their natural counterparts remains less understood, particularly when the restored plantations exhibit varying growth outcomes. This understanding is crucial, as it transcends the mere confirmation that restoration alters microbial communities, aiming instead to identify whether specific microbial assemblages are linked to successful restoration outcomes.

*Kandelia obovata* is a dominant mangrove species along the coastlines of South China and is extensively utilized in restoration projects. This study aims to address the existing knowledge gap by conducting a comparative analysis of the rhizosphere bacterial communities of *Kandelia obovata* in a natural forest and two artificially restored forests with contrasting growth statuses (well-grown and poorly-grown). We hypothesize that artificial restoration does not necessarily alter the overall bacterial community structure but selectively enriches specific bacterial taxa, and that the presence or absence of these key taxa is correlated with the growth performance of *Kandelia obovata*.

By employing high-throughput sequencing of the 16 S rRNA gene, this study aims to clarify several key aspects. First, we will compare the alpha and beta diversity of rhizosphere bacteria between natural and artificially restored *Kandelia obovata* forests. Second, we will identify differentially enriched bacterial taxa, particularly between artificially restored sites that exhibit contrasting plant growth performance. Lastly, we will discuss the potential ecological roles of these key taxa in the context of successful mangrove restoration. Our findings will provide a theoretical basis for utilizing microbial indicators to assess and enhance the outcomes of mangrove afforestation efforts.

## Methods

### Research locations

*Kandelia obovata* specimens were collected from three distinct locations for this study. Natural samples were collected from a 200-meter diameter area centered at 21.739° N, 108.648° E in the Jingu River, representing naturally growing populations of *Kandelia obovata*. The treat-k samples were obtained from a 200-meter diameter area centered at 21.772° N, 108.659° E in Kongque Bay, where *Kandelia obovata* seedlings had been cultivated through artificial restoration. The treat-s samples were collected from Shuijingkeng, within a 200-meter radius centered at 21.739° N, 108.621° E, an area that had also undergone artificial restoration of *Kandelia obovata* seedlings. The study took place in a subtropical monsoon climate zone with an average annual temperature of 22–24 ℃ and an average annual rainfall of 1609–2174 mm. All three locations were situated within the Qinzhou Port Economic Development Zone. The natural area featured a mixed forest of *Aegiceras corniculatum* and *Kandelia obovata*. Treat-k involves converting project land into a *Kandelia obovata* plantation, while treat-s involves transforming a shrimp pond into a plantation of *Aegiceras corniculatum*, *Avicenniamarina* (Forsk.) Vierh. *hailanci*, and *Kandelia obovata*. The forests were in its second year of growth.

### Sample collection

In this study, *Kandelia obovata* seedlings were randomly selected from the sediment, following a modified sampling method based on the literature^9^. Measurements were conducted to assess plant height, diameter at 10 cm and 40 cm from the ground, as well as the width and length of mature leaves. The seedlings were carefully uprooted with loose soil around the roots being removed, and rhizosphere collected and subsequently mixed evenly. The soil was promptly placed into a sampling tube, transferred to liquid nitrogen for 2 h, and stored at -80 ℃ before transportation to Wuhan on dry ice. Microbiological assays were carried out by Wuhan Metware Biotechnology Co.,Ltd (https://www.metware.cn/).

A total of nine independent rhizosphere soil samples were collected for this study, consisting of three biological replicates for each of the three conditions: the natural mangrove forest (natural), the well-developed artificial restoration site (treat-k), and the poorly developed artificial restoration site (treat-s).

The sample was identified by Professor Riming Wang, a PhD in Horticulture, as the roots of *Kandelia obovata*, a species belonging to the genus *Kandelia* in the family Rhizophoraceae. The specimens are currently stored in the Herbarium of the Mangrove Research Institute at Beibu Gulf University, under the catalog numbers Gong_QZ_20230401_001, Gong_QZ_20230401_002, and Gong_QZ_20230401_003.

### Extraction of genome and 16 S rDNA sequencing

Total genome DNA from soil samples was extracted using CTAB method. DNA concentration and purity was monitored on 1% agarose gels. According to the concentration, DNA was diluted to 1ng/µL using sterile water.

The V3-V4 hypervariable regions of the bacterial 16 S rRNA gene were amplified using specific primers 341 F (CCTAYGGGRBGCASCAG) and 806R (GGACTACNNGGGTATCTAAT), which included barcode sequences. All PCR reactions were carried out with 15 µL of Phusion^®^ High-Fidelity PCR Master Mix (New England Biolabs); 2 µM of forward and reverse primers, and about 10 ng template DNA. Thermal cycling consisted of initial denaturation at 98 ℃ for 1 min, followed by 30 cycles of denaturation at 98 ℃ for 10 s, annealing at 50 ℃ for 30 s, and elongation at 72 ℃ for 30 s. Finally 72 ℃ for 5 min. Mix same volume of 1× loading buffer (contained SYBR green) with PCR products and operate electrophoresis on 2% agarose gel for detection. PCR product was mixed in equidensity ratios. Then, mixture PCR product was purified with Qiagen Gel Extraction Kit (Qiagen, Germany).

Sequencing libraries were generated using TruSeq^®^ DNA PCR-Free Sample Preparation Kit (Illumina, USA) following manufacturer’s recommendations and index codes were added. The library quality was assessed on the Qubit@ 2.0 Fluorometer (Thermo Scientific) and Agilent Bioanalyzer 2100 system. At last, the library was sequenced on an Illumina NovaSeq platform and 250 bp paired-end reads were generated.

Paired-end reads were assigned to samples based on their unique barcode and truncated by cutting off the barcode and primer sequence. Quality filtering on the raw tags were performed under specific filtering conditions to obtain the high-quality clean tags according to the fastp (v0.22.0, https://github.com/OpenGene/fastp). Paired-end reads were merged using FLASH (v1.2.11, https://ccb.jhu.edu/software/FLASH/), a very fast and accurate analysis tool, which was designed to merge paired-end reads when at least some of the reads overlap the read generated from the opposite end of the same DNA fragment. The tags were compared with the reference database (Silva database (16 S), https://www.arb-silva.de) using UCHIME Algorithm (https://www.drive5.com/usearch/manual/uchime_algo.html) to detect chimera sequences, and then the chimera sequences were removed. Then the Effective Tags finally obtained.

### Bacteria species annotation

Amplicon sequence variants (ASVs) were analyzed by Deblur, which uses error profiles to　obtain putative error-free sequences from Illumina MiSeq and HiSeq sequencing platforms. For each representative sequence, the Silva Database (https://www.arb-silva.de/) was used based on Mothur algorithm to annotate taxonomic information.

In order to study the phylogenetic relationship of different ASVs and the differences in the dominant species among different samples (groups), multiple sequence alignment were conducted　using the MAFFT(v7.490, https://mafft.cbrc.jp/alignment/software/).

ASVs abundance information was normalized using a standard of sequence number corresponding to the sample with the least sequences. Subsequent analysis of alpha diversity and beta diversity were all performed basing on this output normalized data.

### Data analysis

Alpha diversity is applied in analyzing complexity of species diversity for a sample through 4 indices, including Observed-species, Chao1, Shannon, Simpson et al. All this indices in our samples were calculated with QIIME and displayed with R software (Version 4.1.2). Beta diversity analysis was used to evaluate differences of samples in species complexity, Beta diversity on weighted were calculated by QIIME software. Cluster analysis was preceded by principal component analysis (PCA), which was applied to reduce the dimension of the original variables using the stats package and ggplot2 package in R software. A distance matrix of weighted unifrac among samples obtained before was transformed to a new set of orthogonal axes, by　which the maximum variation factor is demonstrated by first principal coordinate, and the second maximum one by the second principal coordinate, and so on.

Graphic drawing was performed using the Metware Cloud, a free online platform for data analysis (https://cloud.metware.cn/).

We employed Amplicon Sequence Variants (ASVs) for community analysis instead of traditional Operational Taxonomic Units (OTUs). ASVs offer single-nucleotide resolution, which reduces errors and enables more precise differentiation of closely related bacterial taxa across samples. This precision is crucial for identifying fine-scale microbial shifts in response to restoration practices.

### Bacterial co-occurrence network analysis

The bacterial co-occurrence network analysis was conducted using the relative abundance matrix of ASVs at the genus level. We employed the psych package in R (version 4.1.2) to compute the Spearman correlation coefficient, thereby constructing a correlation matrix among species. Only edges with an absolute correlation coefficient of ≥ 0.8 and a p-value of < 0.05 were retained, while self-loops of nodes and connections with node relative abundance < 0.005% were excluded. The network graph was constructed and visualized using the igraph package (version 1.3.5), and the following topological parameters were calculated: network diameter, modularity, average clustering coefficient, graph density, average degree, and average path length. Additionally, key nodes, characterized by high betweenness centrality and high closeness centrality, were identified to evaluate their potential functional roles within the network.

### Functional prediction analysis

Based on 16 S rRNA gene sequencing data, we utilized PICRUSt2 (Phylogenetic Investigation of Communities by Reconstruction of Unobserved States, v2.5.0) to predict the functional potential of bacterial communities. Initially, the ASV representative sequences were aligned against a reference database, such as Greengenes, to generate a phylogenetic tree. Subsequently, the functional composition of each ASV was inferred using precomputed gene family copy numbers. Finally, the predicted results were mapped to the KEGG (Kyoto Encyclopedia of Genes and Genomes) database to determine the relative abundance of each sample at the KEGG pathway level^10^. All analyses were conducted using default parameters, and functional annotation was performed through KEGG Orthology (KO) numbers.

## Results

### Growth status

**Fig. 1:**
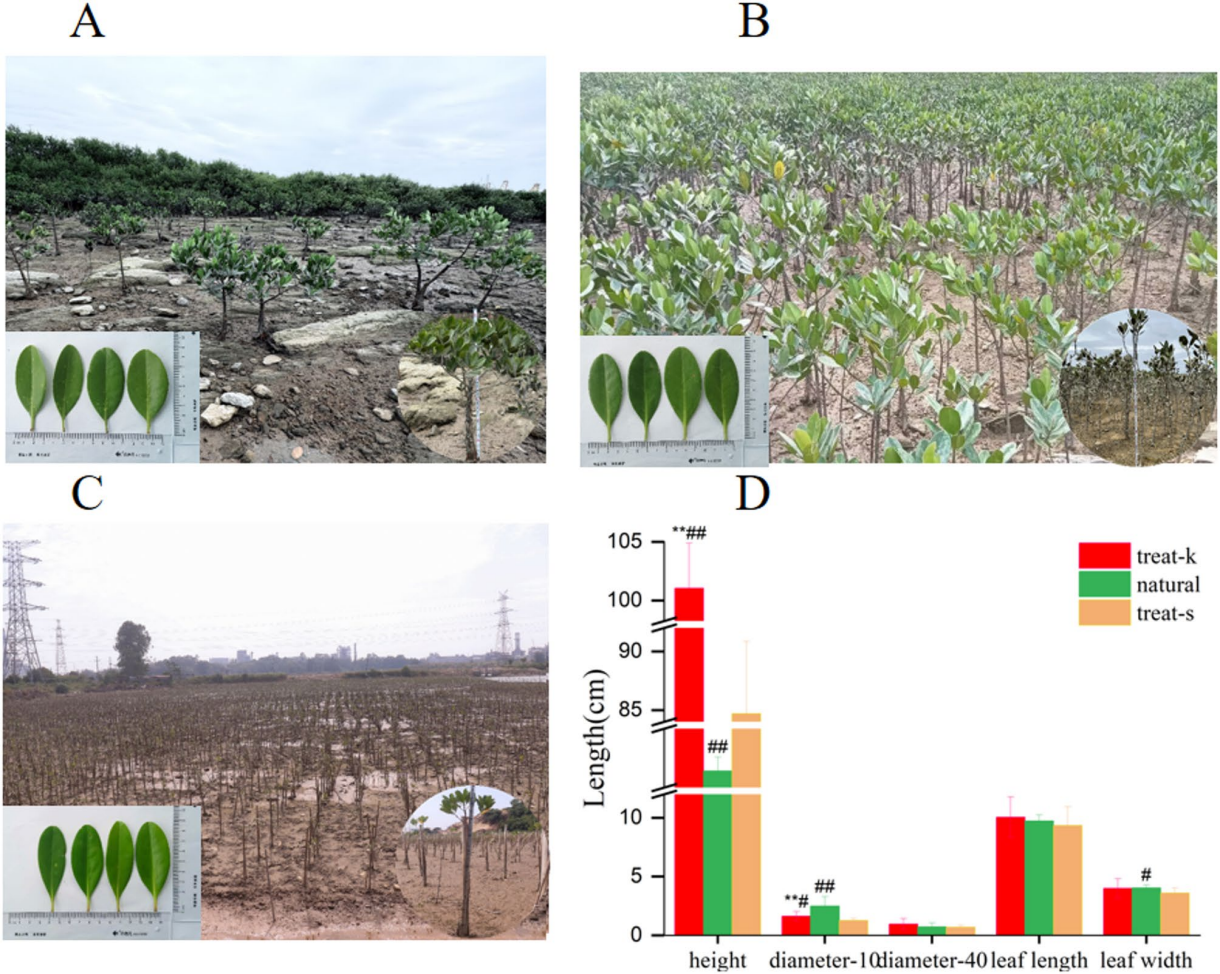
Growth status of *Kandelia obovata* forest. (**A**) Natural growth of *Kandelia obovat*a. (**B**) Treat-k artificial transplantation of *Kandelia obovata*. (**C**) Treat-s artificial transplantation of *Kandelia obovata*. (D) Growth status of *Kandelia obovata*. Height represents the height of *Kandelia obovata*, diameter-10 represents the plant diameter in the height of 10 cm from the ground, diameter-40 represents the plant diameter in the height of 40 cm above the ground, leaf length represents the length of the blade, and leaf width is the width of the blade. vs. natural, * *p* < 0.05, ** *p* < 0.01; vs. treat-s, # *p* < 0.05, ## *p* < 0.01.

Natural *Kandelia obovata* seedlings grow in its natural habitat with sparse plants, thick branches, and large diameters close to the soil. Treat-k seedlings, which are artificially transplanted, exhibit strong growth with a high density of branches and leaves, significantly taller than treat-s and natural seedlings. The diameter at 10 cm above the ground is also larger than treat-s seedlings, although smaller than natural seedlings. Treat-k seedlings also have larger leaf dimensions compared to treat-s and natural seedlings. In contrast, treat-s seedlings grow sparsely with shorter height, smaller diameter, leaf length, and width. The differences in growth parameters between treat-k and treat-s seedlings are statistically significant at certain heights, while natural seedlings generally exhibit larger dimensions across the board (Fig. 1). Figure 1A and B, and 1C present the morphological appearance diagrams of *Kandelia obovata* from various groups, with Fig. 1D displaying the corresponding statistical bar chart.

### Bacteria complexity analysis

**Fig. 2:**
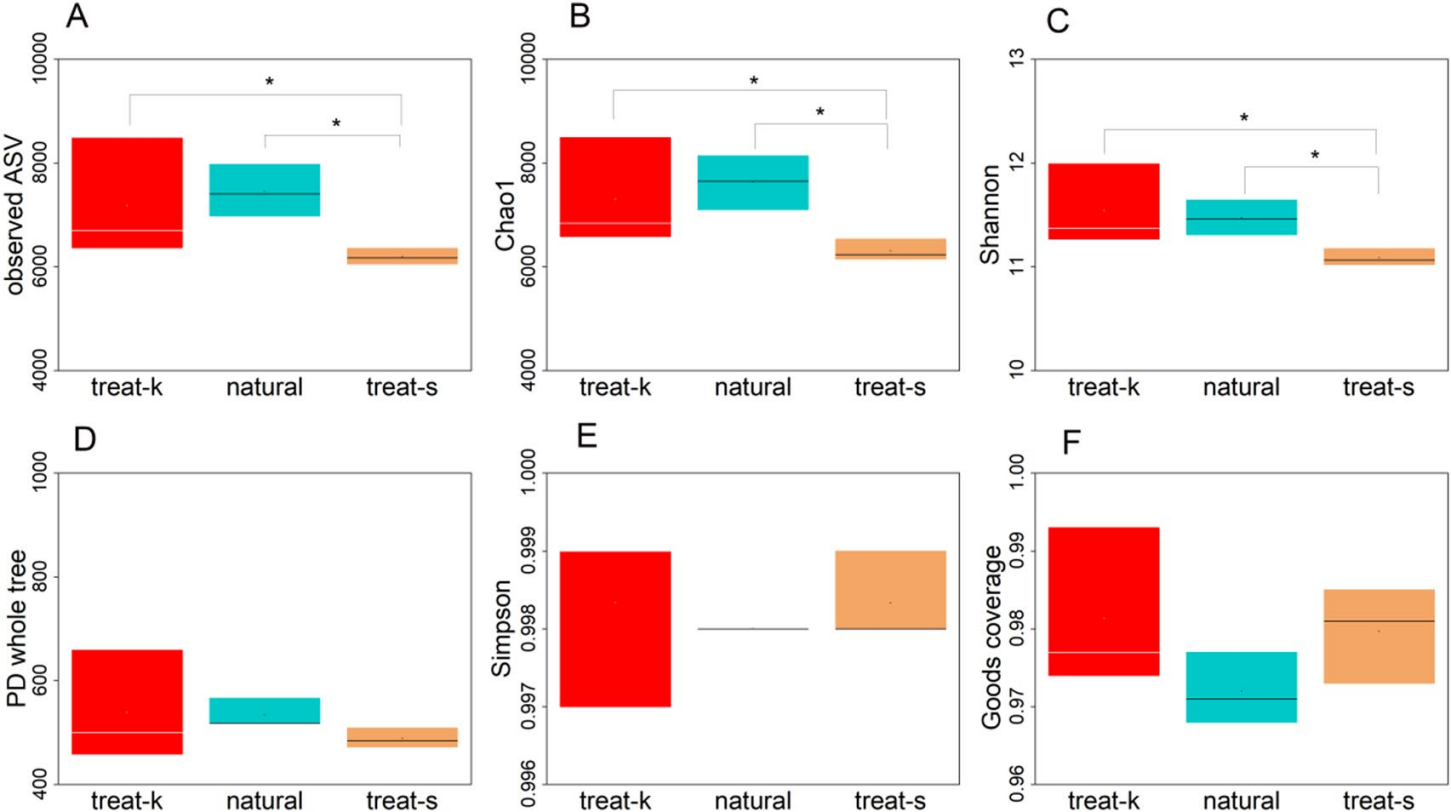
Analysis of bacterial alpha-diversity. (**A**) Observed ASVs; (**B**) Chao1 index; (**C**) Shannon index; (**D**) Faith’s PD index; (**E**) Simpson index; (**F**) Good’s coverage.

The alpha analysis of ASV revealed that the three samples exhibited some similarities, as well as notable differences. Figure 2 presents a box plot illustrating the graphical representation of the alpha analysis of soil bacteria in the rhizosphere of *Kandelia obovata.* Analysis of the Observed ASV index indicated that the treat-s group was significantly different from both the natural group and the treat-k group; however, no significant difference was observed between the natural group and the treat-k group (Fig. 2A). Similarly, the Chao1 index (Fig. 2B), which reflects bacterial richness, and the Shannon index (Fig. 2C), which indicates bacterial diversity, also demonstrated comparable significant results, while the Simpson index did not reveal any significant differences (Fig. 2E). Additionally, the PD whole tree index (Fig. 2D) and the Goods coverage index (Fig. 2F) did not show significant differences.

### Bacteria species differences

LEfSe (LDA Effect Size) analysis was performed on ASV samples, identifying 366 species of bacteria across different groups with statistically significant differences (*p* < 0.05). This comprised 91 species in treat-k, 119 species in natural samples, and 156 species in treat-s, accounting for 24.9%, 32.5%, and 42.6% respectively.

**Fig. 3:**
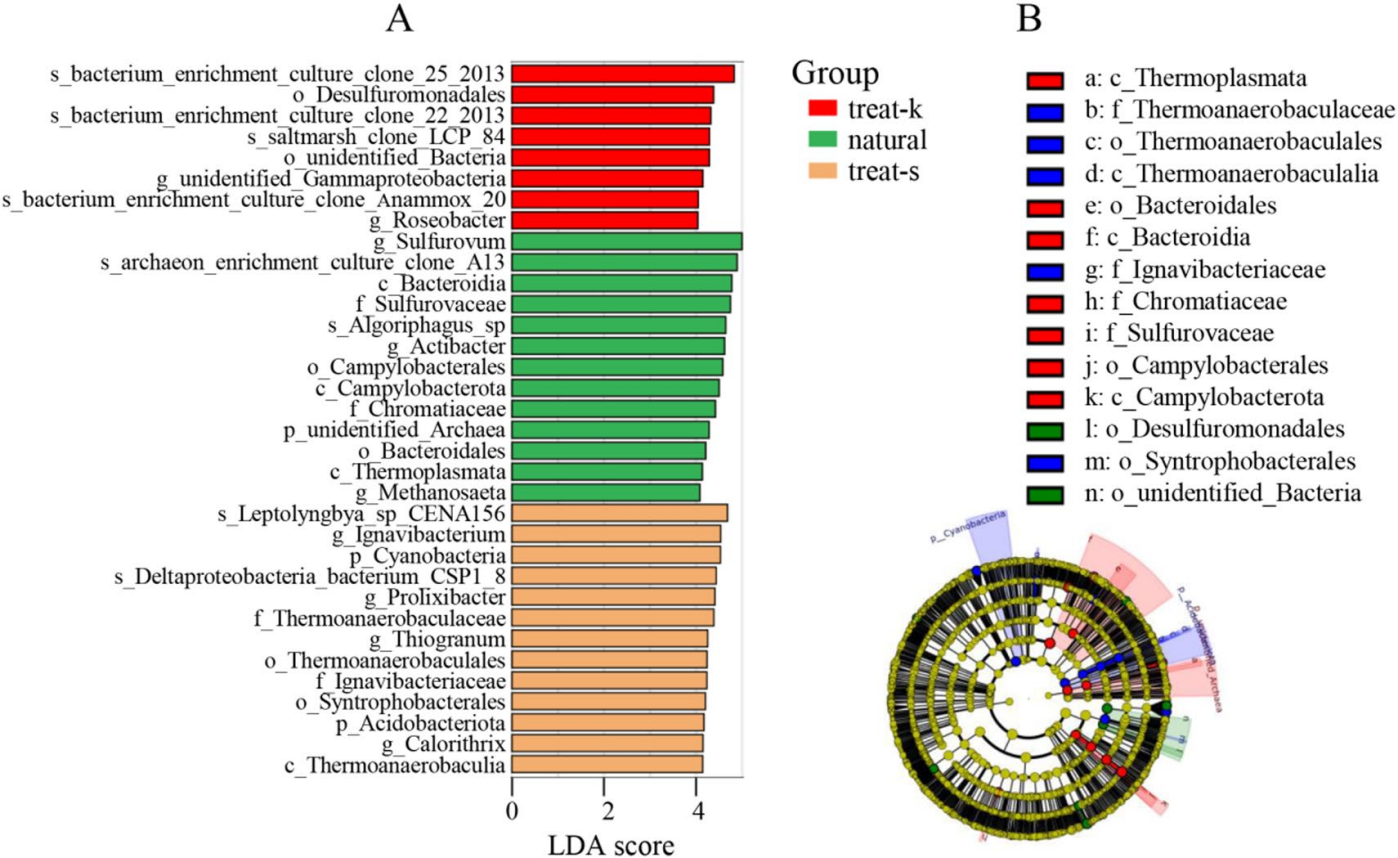
Analysis of bacteria differences. (**A**) LEFse score barplot; (**B**) LEFse cladogram.

In the treat-k sample, taxa such as o-Desulfuromonadales, *Roseobacter*, s-bacterium enrichment culture clone Anammox20, s-saltmarsh clone LCP84, and s-bacterium enrichment culture clone22.2013 exhibit statistically significant differences, with LDA scores ≥ 4.0 (Fig. 3A).

In natural samples, taxa such as c-Bacteroidia, c-Campylobacterota, c-Thermoplasmata, o-Campylobacterales, o-Bacteroidales, f-Chromatiaceae, f-Sulfurovaceae, *Actibacter*, *Methanosaeta*, *Sulfurovum*, and s-archaeon enrichment culture clone A13 show statistically significant differences and may have a dominant role among bacteria (Fig. 3A).

In treat-s samples, p-Cyanobacteria、p-Acidobacteriota、c-Thermoanaerobaculia、o-Thermoanaerobaculales、o-Syntrophobacterales、f-Thermoanaerobaculaceae、f-Ignavibacteriaceae、*Thiogranum*、*Calorithrix*、*Ignavibacterium*、*Prolixibacter*、s-Deltaproteobacteria bacterium CSP1.8、s-Leptolyngbya sp CENA156 display statistically significant differences (Fig. 3B).

### Multiple sample comparison

**Fig. 4:**
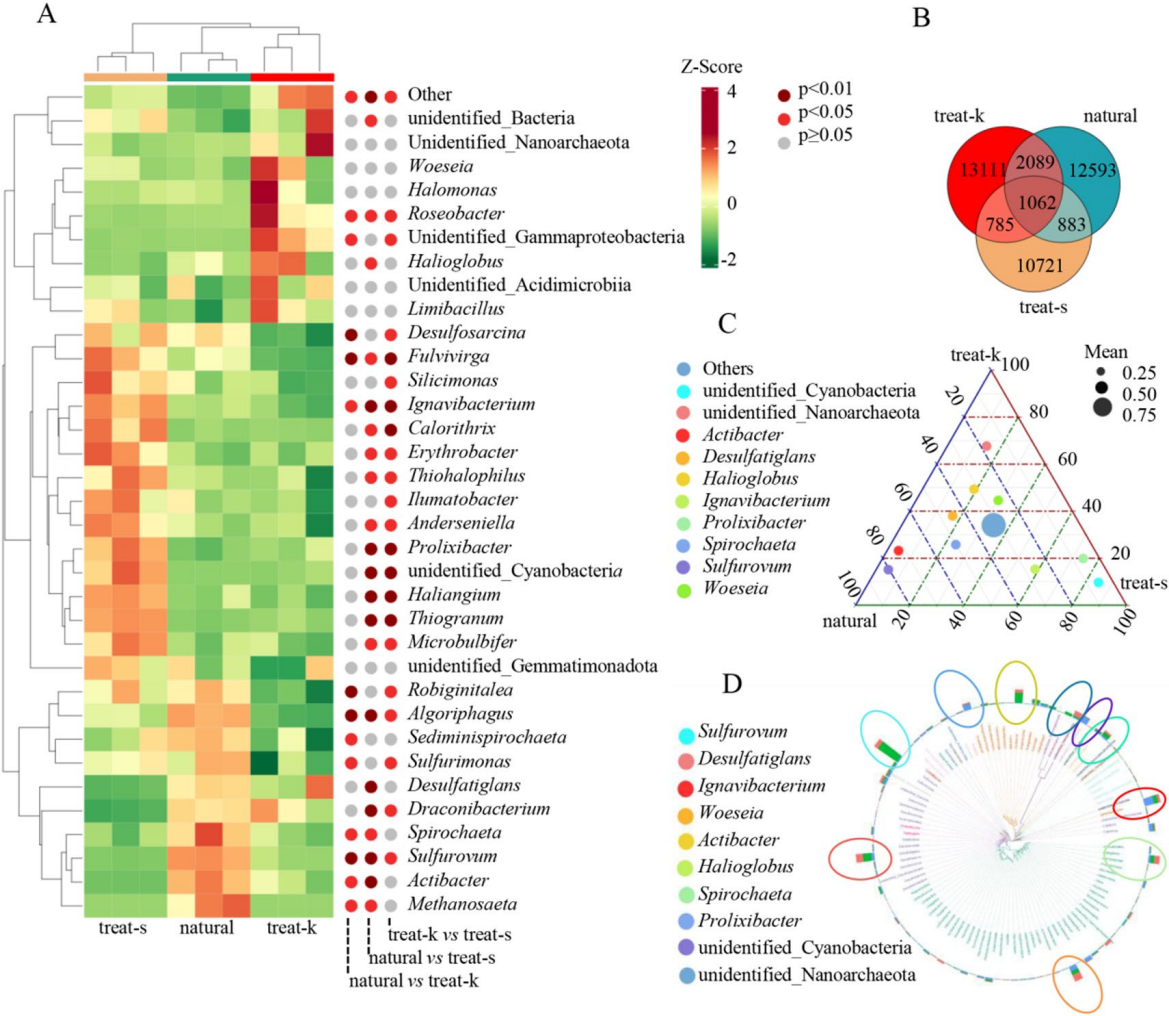
Bacteria analysis based on ASV. (**A**) Quantitative data heat map and Metastats significant difference identification map; (**B**) Venn diagram; (**C**) Ternary phase diagram; (**D**) Genus level species system diagram.

Cluster analysis indicated that samples treat-k were more similar to natural samples, showing a slight difference in species diversity (Fig. 4A). Venn diagram analysis reveals that the numbers of bacteria taxa identified by treat-k, natural, and treat-s are 17,047, 16,621, and 13,451 respectively (Fig. 4B). The numbers of individual characteristic bacteria are 13,111, 12,593, and 10,721 respectively. The dominant bacteria in the rhizosphere soil of *Kandelia obovata* in mangrove forests included *Sulfurovum*, *Actibacter*, *Desulfatiglans*, *Ignavibacterium*, *Woeseia*, *Spirochaeta*, *Halioglobus*, *Robiginitalea*, *Sulfurimonas*,* Prolixibacter*, and *Algoriphagus*. Among these, *Sulfurovum*, *Ignavibacterium*, *Prolixibacter*, and *Algoriphagus* not only had high abundance but also displayed significant differences between groups (Fig. 4C, D).

### Bacterial community

**Fig. 5:**
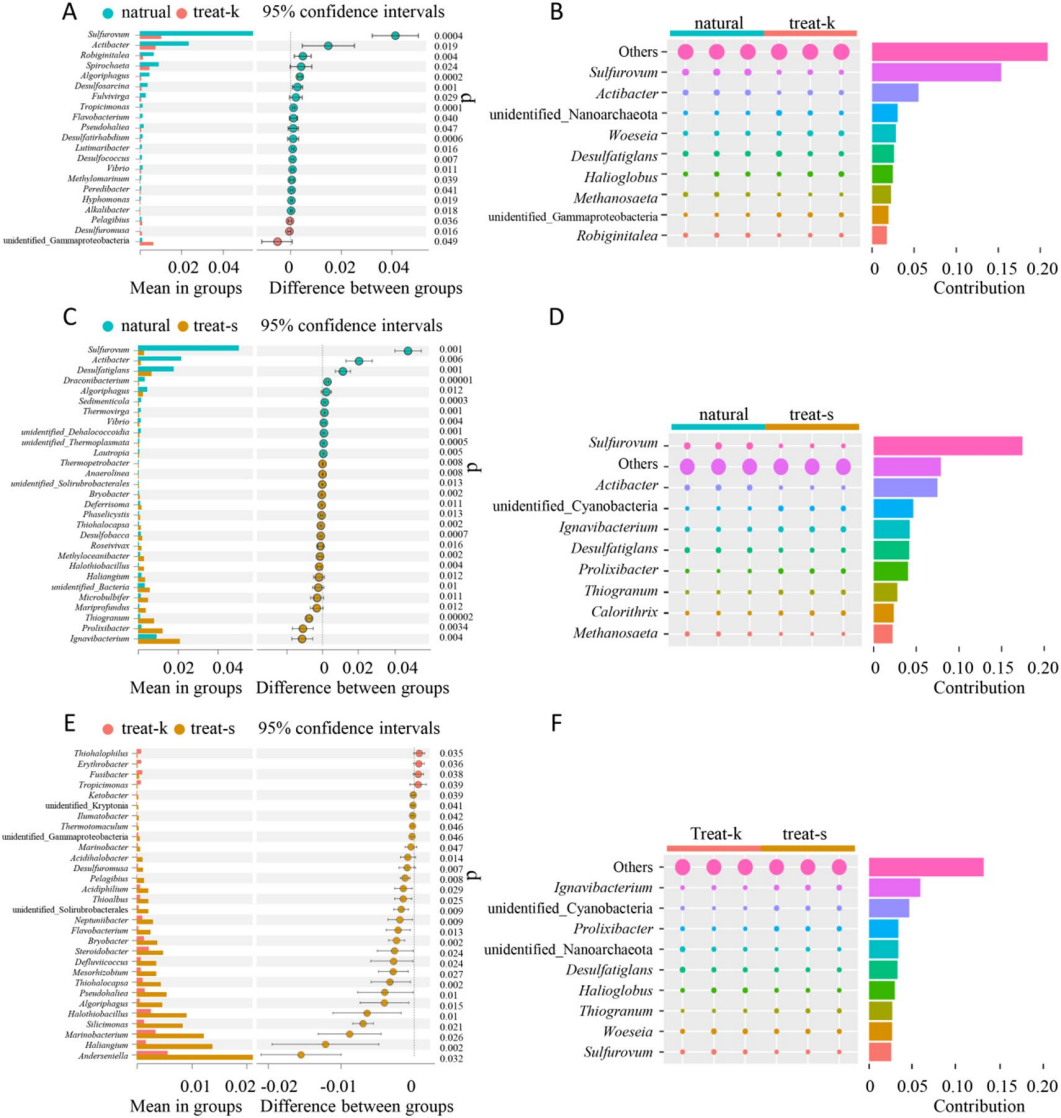
Bacteria significance analysis. (**A**,** C**,** E**). ASV-based T-test species difference analysis chart between groups. (**B**,** D**,** F**). ASV-based Simper differential contribution.

Bacterial contributions to sample differences varied among locations, with the natural sample originating from a natural mangrove, and the treat-k and treat-s samples from artificially restored and planted mangroves. Variations in bacteria were observed, particularly with *Actibacter* and *Methanosaeta* genera showing significant differences in abundance between natural samples and those from the other locations (*p* < 0.01). Furthermore, the *Sulfurovum* showed higher abundance in natural, treat-k, and treat-s samples, with percentages of 5.09%, 0.96%, and 0.29% respectively (Fig. 5A and C), contributing 15.36% to the difference with the treat-k sample and 17.45% to the difference with the treat-s sample (Fig. 5B and D). There was no significant difference in the abundance of bacteria from the genus *Sulfurovum* between treat-k and treat-s samples. In the natural, treat-k, and treat-s samples, the abundance of *Actibacter* bacteria was 2.18%, 0.70%, and 0.13% respectively (Fig. 5A, C and E). When comparing different groups, their respective contribution shares were 5.52% and 7.47% (Fig. 5B, D and F). The abundances of *Methanosaeta* in the three samples were 0.61%, 0.003%, and 0% respectively (Fig. 5A, C and E), with contribution shares in the comparison of different groups at 2.26%, 2.22%, and 0.01% respectively (Fig. 5B, D and F).

### Bacteria interaction network

ASV (amplicon sequence variants) analysis is a valuable tool for studying bacteria communities and their associations using 16 S rDNA sequences. At the genus level, ASVs are classified into 85 known and 16 unidentified bacteria. Some of the most prevalent genera include *Sulfurovum*,* Desulfatiglans*, *Ignavibacterium*, *Woeseia*, *Actibacter*, *Halioglobus*, *Spirochaeta*, *Prolixibacter*.

**Fig. 6:**
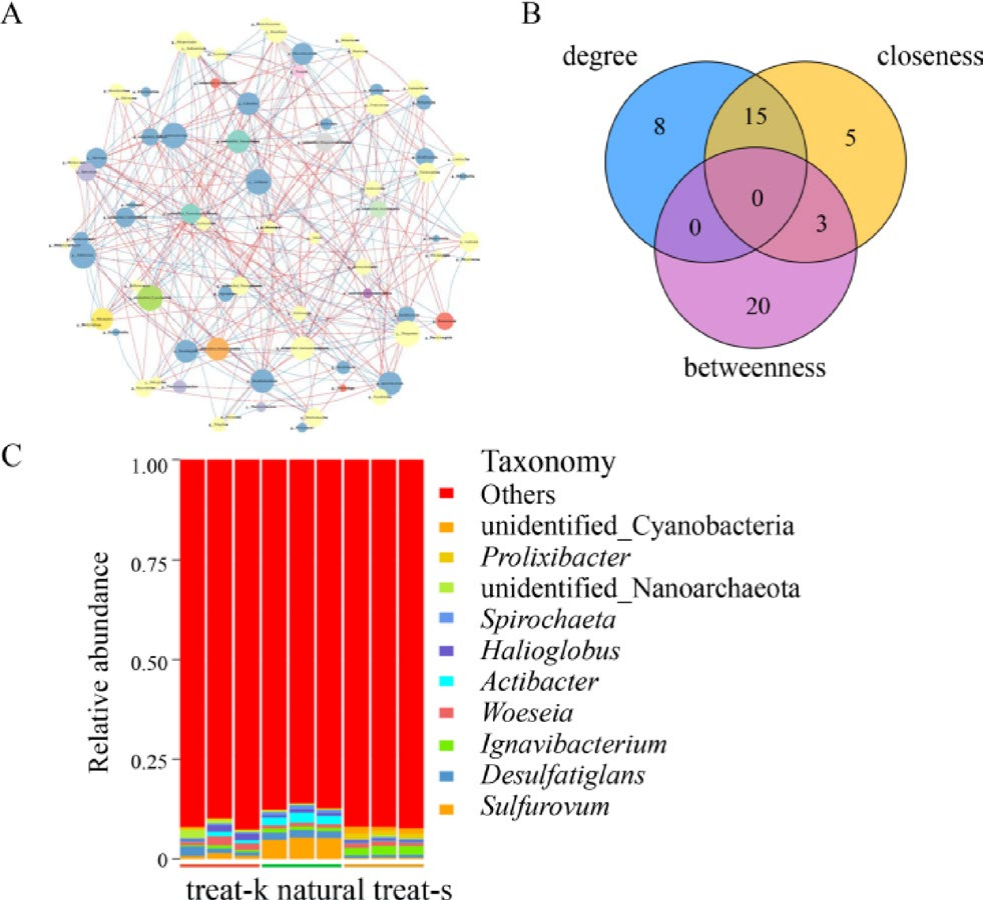
Correlation analysis of bacteria network. (**A**) Spatial network diagram; (**B**) Venn diagram of key species; (**C**) Relative abundance diagram of species at genus level.

Sequences were classified based on abundance levels, and representative sequences of the genera were illustrated in Fig. 6A. The distribution of bacteria varies among different sample groups. In the treat-k sample, bacteria with a higher proportion at the genus level include *Woeseia*, *Desulfatiglans*, *Halioglobus*, *Sulfurovum*, *Actibacter*, *Ignavibacterium*, *Roseobacter*, *Spirochaeta*, *Halomonas*, *Prolixibacter*, *Draconibacterium*, *Calorithrix*, *Limibacillus*, and *Deferrisoma*. Bacteria with an abundance greater than 1% include *Woeseia*, *Desulfatiglans*, and *Halioglobus*, with abundances of 1.56%, 1.53%, and 1.11% respectively. Within the natural group samples, bacteria with higher abundance at the genus level include *Sulfurovum*, *Actibacter*, *Desulfatiglans*, *Ignavibacter*, *Woeseia*, *Spirochaeta*, *Halioglobus*, *Methanosaeta*, *Robiginitalea*, *Algoriphagus*, *Sulfurimonas*, *Sediminispirochaeta*, *Desulfosarcina*, and *Draconibacteria*. Specifically, *Sulfurovum*, *Actibacter*, and *Desulfatiglans* are the bacteria with abundances exceeding 1%, with proportions of 5.09%, 2.18%, and 1.82% respectively. Lastly, in the treat-s samples, bacteria with more content at the genus level include *Ignavibacterium*, *Prolixibacter*, *Woeseia*, *Calorithrix*, *Thiogranum*, *Desulfatiglans*, *Robiginitalea*, *Anderseniella*, *Halioglobus*, *Fulvivirga*, *Microbulbifer*, and *Spirochaeta*. Additionally, bacteria groups with abundances over 1% were observed, such as *Ignavibacterium*, *Prolixibacter*, and *Woeseia*, with abundances of 2.10%, 1.21%, and 1.06% respectively. Unexpectedly, the study found that the dominant bacteria in the rhizosphere soil of two artificial restoration, one natural *Kandelia obovata* forests, were completely different.

The key species exhibit high betweenness centrality, serving as crucial hubs in regulating bacterial functions and playing a significant role in bacterial regulatory processes (Fig. 6A). Despite having relatively low closeness centrality, the efficiency of interaction and communication with other bacteria remains inadequate (Fig. 6B). The top 100 bacteria were selected at the genus level for correlation analysis. Connections with an absolute correlation coefficient ≤ 0.8 were removed, along with node self-connections and connections with node abundance less than 0.005%. The resulting ASV network diagram (Fig. 6C) showed a network diameter (ND) of 8, a modularity coefficient (MD) of 0.52, an average clustering coefficient (CC) of 0.48, a network graph density (GD) of 0.08, an average connectivity (AD) of 6.36, and an average path length (APL) of 3.24.

A small number of bacteria groups were detected in the samples, accounting for only 7.4%-13.9% of the total, while unidentified bacteria made up a larger portion (Fig. 6C).

### Function prediction

**Fig. 7:**
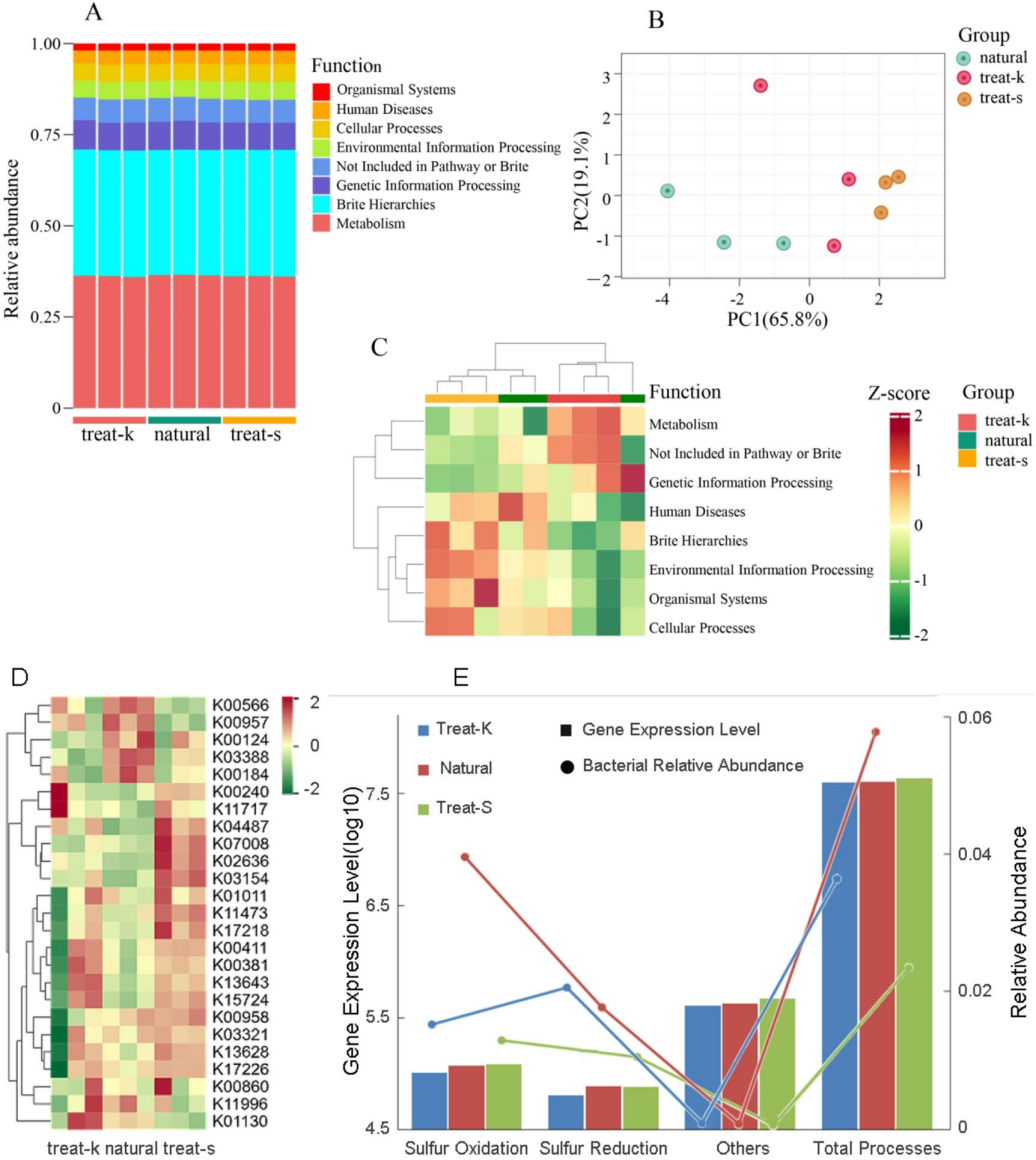
Bacteria functional annotation. (**A**) Functional annotation relative abundance plot; (**B**) Functional annotation principal component analysis plot; (**C**) Functional annotation heat map; (**D**) Heatmap of sulfur metabolism dominant gene expression; (**E**) Comparison of sulfur metabolism genes and abundance.

Functional prediction analysis of 16 S sequencing data was performed using the KEGG database(https://www.kegg.jp/kegg/kegg1.html). The initial results of functional annotation showed that Metabolism and Brite Hierarchies accounted for a larger proportion of functional structures, approximately 36% and 34% respectively, while other groups represented about 1.9% to 7.7% with no significant differences observed between the samples (Fig. 7A). However, PCA analysis of the functional statistical results indicated that principal component 1 and principal component 2 collectively represented 84.9% (Fig. 7B). Cluster analysis, based on functional annotation results and abundance, demonstrated distinct differences between the two artificial restoration bases, with some clustering overlap between samples from the natural forest and the restoration base (Fig. 7C). The expression of key genes in the sulfur metabolism prediction pathway varies among different groups (Fig. 7D). Notably, the treat-k group exhibited the lowest expression levels of various sulfur metabolism genes, whereas the treat-s group demonstrated the highest expression levels in sulfur oxidation, other sulfur-related functions, and overall sulfur metabolism, indicating distinct responses. In terms of the abundance of sulfur metabolism-related bacteria, the treat-s group displayed lower levels of sulfur-oxidizing, sulfur-reducing, and other sulfur metabolism-related bacteria compared to the other groups (Fig. 7E), revealing a significant discrepancy between functional gene expression and bacterial abundance.

## Discussion

### Growth status

The growth and mortality of *Kandelia obovata* seedlings are influenced by a complex interplay of edaphic factors, including the root matrix^11^, soil structure, and chemical composition. In this study, while a comprehensive analysis of soil physicochemical properties across all sites was not conducted, preliminary observations and the proximity of the sites within the same economic zone suggest that major soil structural and compositional parameters, as well as water salinity, were broadly comparable. Given the similarity of these fundamental environmental conditions, the pronounced differences in seedling growth performance, particularly between the two artificially restored sites (treat-k and treat-s), cannot be readily explained by these factors alone. This observation led us to hypothesize that variations in the rhizosphere bacterial community might play a pivotal role in mediating plant growth under these specific conditions. It is well established that bacteria are crucial for nutrient cycling and plant health in mangroves. Furthermore, factors previously reported to influence mangrove growth, such as polycyclic aromatic hydrocarbons, total nitrogen, organic carbon^12^, organic acids^5,13^, and tidal height^14^, often exert their effects by shaping the soil microbial community. Therefore, we propose that the differential enrichment of key bacterial taxa, as identified in our study, is a strong candidate mechanism underlying the contrasting growth status of *Kandelia obovata* in the natural, treat-k, and treat-s forests. Future studies incorporating detailed soil geochemical analyses are warranted to disentangle the direct effects of soil properties from the indirect effects mediated by the microbiome.

### Differential enrichment of bacterial taxa revealed by LEfSe analysis

LEfSe analysis of our ASV data revealed distinct bacterial taxa enriched in the rhizosphere of *Kandelia obovata* under varying growth conditions. This method facilitates the identification of biomarkers that are statistically significant among different groups^15^. A substantial number of bacterial species (366 in total) exhibited significant differences (*p* < 0.05) across the three sample groups: 91 in the treat-k group, 119 in the natural group, and 156 in the treat-s group. Notably, the poorly grown treat-s group harbored the highest number of differentially abundant species, suggesting a potentially more divergent or stressed microbial state. The LDA scores (LDA ≥ 4.0) further identified specific taxa characteristic of each group. For instance, the well-grown artificial group (treat-k) was significantly enriched with taxa such as *Roseobacter*, while the natural forest was characterized by *Actibacter*, *Methanosaeta*, and *Sulfurovum*. In contrast, the poorly grown artificial group (treat-s) was dominated by *Ignavibacterium*, *Prolixibacter*, and *Thiogranum*. This clear partitioning of microbial taxa aligns with the observed plant growth status and underscores the potential functional significance of these enriched bacteria.

### Comparative analysis with other Mangrove ecosystems

In comparison to other mangrove ecosystems, the dominant bacterial genera identified in our study exhibit both commonalities and unique patterns. For instance, a study conducted in the Zhangjiangkou National Mangrove Nature Reserve reported a high abundance of *Staphylococcus* in the rhizosphere of *Kandelia obovata*, a genus that was not prominent in our samples. This discrepancy underscores how local environmental conditions and anthropogenic influences can significantly shape the rhizosphere microbiome. Moreover, while the Zhangjiangkou study observed minimal overall differences in bacterial composition between samples using OTU clustering, our application of higher-resolution ASV analysis successfully detected significant shifts in specific taxa, despite a stable community-level profile. This contrast highlights the advantage of ASV in revealing subtle yet ecologically critical microbial changes that may be overlooked by OTU-based methods.

The primary bacterial genera identified in the vegetation coverage area of the Beidaihe coastal wetland include *Planctomyces*, *Exiguobacterium*, *Citrobacter*, and *Rhodopirellula*, which exhibit significantly higher abundance compared to those in the vegetation-free area. In contrast, the predominant bacterial genera in the vegetation-free area comprise *Desulfosarcina*, *Ilumatobacter*, *Loktanella*, *Actibacter*, and *Sulfurovum*. This study underscores the significant presence of *Sulfurovum* as a key bacterium in mangrove soil, which is consistent with our findings.

*Sulfurovum* is frequently observed in coastal mangrove distribution zones and is a dominant bacterium found on coastal beaches. Our study identified a higher abundance of *Sulfurovum*; however, no significant difference was observed in the abundance of the genus *Sulfurovum* between the two restored *Kandelia obovata* forests. Additionally, *Methanosaeta* and *Actibacter* are also commonly found in coastal mangrove distribution zones and are prevalent bacteria on coastal beaches. Notably, our study revealed significant differences in the abundance of the *Actibacter* and *Methanosaeta* genera, suggesting that these bacteria may serve as potential indicators to differentiate naturally growing mangroves from those that are artificially restored.

### Interaction of characteristic bacteria

The architecture of the bacterial co-occurrence network, derived from our ASV data, identified several keystone taxa based on their positions within the network. Specifically, genera exhibiting high closeness centrality, such as *Halomonas* and *Robertkochia*, are likely to act as influential hubs, a finding that supports the use of network properties to identify functionally important taxa^16^; ^17^. More importantly, the network revealed a significant presence of taxa associated with sulfur metabolism, including Spirochaeta and *Roseobacter*^18^ highlighting the critical role of the sulfur cycle in the biogeochemical processes of the mangrove rhizosphere. This functional insight, derived directly from the network structure, transcends a mere inventory of abundant genera and offers a mechanistic hypothesis for how microbial interactions may underlie ecosystem functioning.

At the genus level, the main rhizosphere bacteria identified in this study were *Sulfurovum*, *Actibacter*, *Woeseia*, and *Halioglobus*. In contrast, the bacteria found in the Zhangjiangkou Mangrove National Nature Reserve mainly consisted of *Alcaligenes*, *Methylobacterium*, *Vibrio*, *Rhodococcus*, *Nocardioides*, *Pseudomonas*, *Actinomycetospora*, *Staphylococcus*, and *Salinisphaera*^19^. It is important to note, however, that a significant portion of the bacterial population in the mangrove rhizosphere remained unidentified in the both results^19^. But their role in the interaction network seems to be limited. These bacteria are likely more focused on material metabolism at an individual level rather than impacting the structure of the surrounding bacteria community.

### Function of characteristic bacteria

Bacteria functions varied to some extent among samples, likely due to differences in bacteria abundance rather than bacteria types. The bacteria of various genera, such as *Draconibacterium*^20^, *Prolixibacter*^21^, *Thiogranum*^22^, *Haliangium*, *Erythrobacter*^23^, *Calorithrix*^24^, *Thiohalophilus*^25^, and *Microbulbifer*^26^, *Prolixibacter*, *Haliangium*, *Erythrobacter*, *Draconibacterium*, *Microbulbifer*, *Thiogranum*, *Thiohalophilus*, *Calorithrix*, *Anderseniella* exhibited varying abundance across different samples.

These bacteria, commonly found in tidal flats, estuaries, and sedimentary layers of mangroves, play a crucial role in metabolizing nitrogen, sulfur, metals, organic pollutants, and polymer plastics^27^. The bacteria *Sulfurovum*, *Ignavibacterium*, *Prolixibacter*, *Algoriphagus*, *Silicimonas*, and *Ilumatobacter*, which show high abundance and significant differences between groups, could potentially impact the growth of *Kandelia obovata*. In high-salinity waters, *sulfurovum* have been observed effectively breaking down benzo(a)pyrene compounds^28^. These bacteria are commonly found in soils near urban areas or in polluted mangrove understories, which are areas affected by pollutants^29^. The composition and level of pollution have a noticeable influence on the structure and abundance of bacteria^12^; ^28^; ^30^. The research was conducted in a natural riverside tidal flat where sediment accumulates in the water. Soil components, such as metal ions and organic matter, are significantly influenced by the water. The study focused on research sites designated for forestland restoration, where the soil has been deeply plowed and filled, resulting in lower pollution levels compared to the water environment. The metal ions and organic matter in the soil are greatly influenced by the components of the landfill, impacting the abundance of each sample. Bacteria such as *Sulfurovum*, *Methanosaeta*, and *Actibacter* are commonly found in coastal mangrove distribution zones.

Natural riverside tidal flats are characterized by sediment accumulation from the soil into the water body, where components such as metal ions and organic matter are significantly influenced. The restored woodlands that have undergone deep plowing and landfilling. The water environmental pollution level is relatively low, but the soil’s metal ions and organic matter are heavily impacted by buried earth components, affecting the abundance of each sample. Common bacteria like *sulfurovum*^31^, *Methanosaeta*^32^; ^33^, and *Actibacter*^34^ are frequently found in coastal mangrove distribution zones, playing a crucial role in metabolizing metals, organic matter, and sulfur-containing compounds along coastal beaches.

Additionally, *Silicimonas*, which is isolated from the cell surface of the diatom *Thalassiosira delicatula*^35^, is likely closely associated with silicon metabolism. The high abundance of *Silicimonas* in the treated sample indicates a significant silicate content in the construction waste present in the soil, which could hinder the growth of *Kandelia obovata*. This hindrance may impede the colonization and development of *Kandelia obovata*. Therefore, it is essential to consider the silicate content in construction waste when restoring coastal beaches to minimize any negative impact on mangrove rehabilitation efforts. Furthermore, *Ilumatobacter* has shown the ability to metabolize persistent organic pollutants such as antibiotics^36^; ^37^ and benzo[a]pyrene (BaP)^38^, potentially aiding in the breakdown of aromatic compounds. This collaborative process helps in the removal of organic matter from the environment^39^.

Functionally classified, sulfur metabolism genes encompass sulfur oxidation, sulfur reduction, sulfur transfer, organic sulfur metabolism, as well as iron-sulfur cluster assembly and auxiliary genes. The key metabolic pathways of the sulfur cycle exhibit significant differences in expression across various sample groups, as illustrated in Fig. 7D. In the natural and treat-k groups, where *Sulfurovum* and *Sulfurimonas* are abundant, the expression of genes related to sulfur oxidation is notably stronger. Conversely, the sulfur reduction process predominantly involves sulfate reduction, with its key genes demonstrating the highest expression in the Natural group, which corresponds with the high abundance of *Desulfatiglans* in this group. The distribution patterns of pathways such as sulfur oxidation and sulfate reduction corroborate the findings of microbial community composition. In the treat-s group, representative genes of sulfur oxidation (such as K17218, K17222–17227, K17225) exhibited elevated expression levels, while the overall expression of genes related to other sulfur metabolic processes (including sulfur transfer, organic sulfur metabolism, and iron-sulfur cluster assembly) surpassed that of both sulfur oxidation and sulfur reduction pathways, indicating a degree of functional overcompensation. The observed inconsistency between gene expression and bacterial abundance in the Treat-s group may be attributed to several mechanisms. Under stress conditions such as sulfide toxicity and hypoxia, residual sulfur-metabolizing bacteria upregulate single-cell activity and highly express key genes. Simultaneously, 86.1%–92.6% of unclassified bacteria may undertake partial sulfur metabolism functions, and various non-specific generalist bacteria may co-express sulfur metabolism genes, forming functional redundancy that maintains metabolic resilience at the community level. The rhizosphere microenvironment of different treatment groups exhibited distinct sulfur cycling states: the natural group maintained an equilibrium state that supports healthy sulfur cycling and plant growth; the treat-k group demonstrated good growth but with slightly diminished sulfur metabolism functions; the treat-s group encountered a more challenging environment, where sulfide accumulation could potentially be toxic to *Kandelia obovata*, and the metabolic hyperactivity observed in its sulfur oxidation process reflects the functional adaptation of the microbial community to environmental stress.

### Bacteria in *Kandelia obovata* forest

The analysis revealed a wide range of distinct bacteria between natural *Kandelia obovata* and restored *Kandelia obovata*, such as *Algoriphagus*, *Sulfurovum*, *Ignavibacterium*, *Actibacter*, *Fulvivirga*, *Roseobacter*, *Spirochaeta*, *Methanosaeta*, and others.

The common significant bacteria in natural environments compared to restored environments and between the both restored samples include *Ignavibacterium*, *Prolixibacter*, *Haliangium*, *Thiogranum*, *Fulvivirga*, *Calorithrix*, *Algoriphagus*, *Sulfurovum*, *Draconibacterium*, *Roseobacter*, *Erythrobacter*, *Thiohalophilus*, *Anderseniella*, *Microbulbifer*, and 14 other species. These bacteria may vary and impact the growth of *Kandelia obovata*.

The genera *Sulfurovum*, *Actibacter*, *Desulfatiglans*, *Ignavibacterium*, *Woeseia*, *Spirochaeta*, *Halioglobus*, *Robiginitalea*, *Sulfurimonas*, *Prolixibacter*, and *Algoriphagus* were identified as more abundant in the sample, indicating their dominance among the bacteria present. Specifically, *Sulfurovum*, *Ignavibacterium*, *Prolixibacter*, and *Algoriphagus* not only displayed high abundance but also exhibited significant differences in key bacteria that could potentially impact the growth status of *Kandelia obovata*.

It is hypothesized that certain bacteria such as *Halomonas*, *Robertkochia*, *Thiogranum*, and *Carboxylicivirga* could be pivotal in bacteria interactions, while others like *Sulfurovum*, *Actibacter*, *Woeseia*, and *Halioglobus* may primarily contribute to the metabolic environment.

It is noted that despite being highly abundant, *Sulfurovum* does not appear to be a central player in network interactions, potentially only participating in material metabolism.

### Limitations and future perspectives

It is crucial to acknowledge the limitations of this study. The primary limitation is the relatively small sample size (*n* = 3 per group), which may constrain the statistical power to detect more subtle differences in the bacterial community structure and increase the susceptibility of our findings to the influence of individual sample variations. Nevertheless, despite this constraint, we identified statistically significant shifts in the abundance of several key bacterial genera, suggesting that the observed effects are substantial. Future investigations that incorporate larger sample sizes and longitudinal sampling across multiple seasons are warranted to validate these findings and to more robustly capture the comprehensive dynamics of rhizosphere microbiota in response to mangrove restoration.

## Conclusion

In artificial *Kandelia obovata* forests, bacteria exhibit similar higher abundance and community structure characteristics. The majority of genera, including *Woeseia*, *Desulfatiglans*, *Halioglobus*, *Spirochaeta*, *Sulfurovum*, *Sulfurimonas*, *Sediminispirochaeta*, *Methyloceanibacter*, *Thermoanaerobaculum*, *Truepera*, *Limibacillus*, *Desulfobacca*, *Actibacter*, *Halomonas*, and *Caldithrix*, did not show significant differences in bacterial abundance between samples.

There is a noticeable difference in the abundance of bacteria found in the rhizosphere soil of vigorously growing and poorly growing saplings within the *Kandelia obovata* forests. Some bacteria, such as *Ignavibacterium*, *Prolixibacter*, *Haliangium*, *Thiogranum*, *Fulvivirga*, *Calorithrix*, *Algoriphagus*, *Sulfurovum*, *Draconibacterium*, *Roseobacter*, *Erythrobacter*, *Thiohalophilus*, *Anderseniella*, and *Microbulbifer*, might play a significant role in influencing the growth of *Kandelia obovata* saplings.

The genera *Sulfurovum*, *Actibacter*, *Desulfatiglans*, *Ignavibacterium*, *Woeseia*, *Spirochaeta*, *Halioglobus*, *Robiginitalea*, *Sulfurimonas*, *Prolixibacter*, and *Algoriphagus* were identified as the dominant bacteria in the samples. Among these, *Sulfurovum*, *Ignavibacterium*, *Prolixibacter*, and *Algoriphagus* stand out for their high abundance and significant differences in key bacteria that could potentially impact the growth status of *Kandelia obovata*.

## Data Availability

The raw read sequences have been deposited in GenBank (NCBI) under the Accession Number PRJNA1252642. They can be accessed through the following link: [https://www.ncbi.nlm.nih.gov/sra/PRJNA1252642](https:/www.ncbi.nlm.nih.gov/sra/PRJNA1252642) .
